# Predicted protein interactions of IFITMs may shed light on mechanisms of Zika virus-induced microcephaly and host invasion

**DOI:** 10.12688/f1000research.9364.2

**Published:** 2017-11-21

**Authors:** Madhavi K. Ganapathiraju, Kalyani B. Karunakaran, Josefina Correa-Menéndez

**Affiliations:** 1 Intelligent Systems Program, University of Pittsburgh, Pittsburgh, PA, USA; 2 Department of Biomedical Informatics, University of Pittsburgh, Pittsburgh, PA, USA; 3 Supercomputer Education and Research Centre, Indian Institute of Science, Bangalore, India

**Keywords:** Zika, virus infection, protein interaction, interferon-inducible transmembrane proteins

## Abstract

After the first reported case of Zika virus (ZIKV) in Brazil, in 2015, a significant increase in the reported cases of microcephaly was observed. Microcephaly is a neurological condition in which the infant’s head is significantly smaller with complications in brain development. Recently, two small membrane-associated interferon-inducible transmembrane proteins (IFITM1 and IFITM3) have been shown to repress members of the flaviviridae family which includes ZIKV. However, the exact mechanisms leading to the inhibition of the virus are yet unknown. Here, we assembled an interactome of IFITM1 and IFITM3 with known protein-protein interactions (PPIs) collected from publicly available databases and novel PPIs predicted using the High-confidence Protein-Protein Interaction Prediction (HiPPIP) model. We analyzed the functional and pathway associations of the interacting proteins, and found that there are several immunity pathways (toll-like receptor signaling, cd28 signaling in T-helper cells, crosstalk between dendritic cells and natural killer cells), neuronal pathways (axonal guidance signaling, neural tube closure and actin cytoskeleton signaling) and developmental pathways (neural tube closure, embryonic skeletal system development) that are associated with these interactors. Our novel PPIs associate cilia dysfunction in ependymal cells to microcephaly, and may also shed light on potential targets of ZIKV for host invasion by immunosuppression and cytoskeletal rearrangements. These results could help direct future research in elucidating the mechanisms underlying host defense to ZIKV and other flaviviruses.

## Introduction

Zika virus (ZIKV) is a flavivirus that was initially isolated from rhesus monkeys in 1947 and was first reported in humans in 1952 ^1^ The flavivirus genus contains around 70 viruses belonging to the family *Flaviviridiae*, a family of positive sense, single-stranded, enveloped RNA viruses ^2^, that also include Dengue virus, West nile virus (WNV), tick-borne encephalitis virus, Japanese encephalitis virus, yellow fever virus, and Hepatitis C virus ^3^. Until recently, ZIKV reports had been limited to Africa and Asia ^4^ but it became a wide-spread ZIKV epidemic ^5^. The virus spread rapidly across the Americas and was declared a ‘global emergency’ by the World Health Organization ^6^. It is mostly transmitted by mosquitoes and clinical manifestations include rash, mild fever, arthralgia, conjunctivitis, myalgia, and headaches. In addition, the virus can be transmitted sexually, with the risk of infection persisting for several months after initial contact ^7^. While the symptoms of ZIKV can be mild ^1^, the virus has been linked to two more serious afflictions: Guillen-Barré syndrome (GBS) ^8, 9^ and microcephaly ^10– 14^, both of which are serious neurological conditions. Microcephaly results in reduced head circumference measurement in infants, exhibiting complications in brain development. Of particular concern is the attribution of microcephaly to infection with ZIKV occurring between the first two trimesters of pregnancy ^13, 14^. Evidence linking ZIKV to microcephaly includes detection of ZIKV RNA in tissue such as the placenta and amniotic fluid of pregnant women with ZIKV, as well as in the brains of stillborn infants with microcephaly ^15^. In a study with human induced pluripotency stem cells, the mechanism of ZIKV related cell death has been elucidated. This study demonstrated that ZIKV infects human embryonic cortical neural progenitor cells (hNPCs), ultimately leading to attenuated population growth mediated by virally induced caspase-3-mediated apoptosis and cell-cycle dysregulation ^16^. Furthermore, mice studies showed that ZIKV infection can lead to nerve degeneration, softening of the brain and porencephaly ^17^. Additional studies have assessed the causal relation between ZIKV infection and birth defects, and the role of ZIKV as a cause of congenital defects and as a trigger of GBS has been established ^10, 18, 19^.

Recently, it was discovered that two small membrane-associated interferon inducible transmembrane proteins (IFITMs) IFITM1 and IFITM3 play a protective role against ZIKV infection by inhibiting replication of the virus and preventing cell death ^7^. The IFITM protein family belongs to a group of small (10 – 15kDa) ^20^ interferon stimulated genes (ISGs), which are in turn produced by the interferon system ^21^. The interferon system is the host’s primary response against infection which restricts entry and fusion from late endosomes ^22^, with some ISGs being involved in suppressing the early stages of viral replication ^21, 23, 24^. IFITMs are located at the cell plasma and endosomal membranes, which are the means of entry of many viruses ^25^. This protein family protects the host against viral infection by directly restricting entry and fusion from late endosomes ^26^.

Flaviviruses depend on the intracellular membrane of their host throughout their lifecycle ^27– 29^. Mice that are lacking immune sensors, signaling pathways and effector molecules were found to be susceptible to flavivirus infection highlighting the importance of the host’s innate immune response to these viruses ^5, 30, 31^. Recent literature has demonstrated that during ZIKV infection, the production of several types of interferon and ISGs is increased, and that IFITM1 and IFITM3 are involved in inhibiting ZIKV infection during the early stages of pathogenesis ^7, 30– 33^. Enveloped viruses, such as ZIKV, have a membrane of host cell lipids that contains viral fusion proteins; these proteins mediate fusion between the virus and the endosomal membrane of the host cell, a necessary step for initiating infection ^21, 34^. The precise mechanisms by which IFITMs inhibit infection are yet to be described. However, IFITM3 has been shown to impact the function of vesicle-associated membrane protein (VAMP)-associated protein A (VAPA) and oxysterol-binding protein (OSBP) by directly interacting with VAPA. This leads to the accumulation of cholesterol in multivesicular bodies and late endosomes, preventing the fusion of the intraluminal virion-containing vesicles with endosomal membranes ^35^. Additionally, IFITM1 has been shown to suppress cell to cell fusion (syncytia formation) by localizing to the plasma membrane after interferon induction ^21^.

There are additional reasons to explore the role of IFITMs in ZIKV restriction. For instance, murine models of ZIKV infection require deficiency of type 1 interferon signaling, which suggests a role for ISGs in restricting infection ^7, 36^. Therefore, prior to the induction of ISGs, IFITMs may provide initial defense against the infection ^7^. Furthermore, IFITMs have been found to restrict virus replication among other flaviviruses, including West Nile virus and Dengue virus ^23, 37, 38^. Recently, the role of IFITM3 as an antiviral protein against West Nile virus has been explored *in vivo*, and *Ifitm3 ^-/-^* mice exhibited a greater susceptibility to lethal viral infection, with a greater accumulation of viral protein in peripheral organs and central nervous system tissues ^26^. Additionally, IFITM1, IFITM2, and IFITM3 have been shown to have antiviral properties in humans. Polymorphisms in human IFITM3 correlate with the severity of influenza A infection ^38^. These functional members are expressed across a variety of tissues in humans and out of the three, IFITM3 is thought to be the most potent line of defense against viral infection ^21^.

As the mechanism through which IFITM1 and IFITM3 mediate restriction is unknown, computational methods could accelerate research by presenting testable hypotheses. Protein-protein interactions (PPIs) prove to be valuable in understanding the function of a protein, and specifically in how it plays a role in causing or preventing disease. Motivated by this, we had developed a computational model called ‘High-confidence Protein-Protein Interaction Prediction’ (HiPPIP) model that identifies novel PPIs in the human interactome ^39^ using machine learning to classify features of protein-pairs such as colocalization, coexpression, shared molecular function and biological processes. *HiPPIP* was also instrumental in discovering that *oligoadenylate synthetase like* protein (OASL) interacts with *retinoic acid inducible gene I* product (RIG-I) to activate the RIG-I immunity pathway during influenza viral infection inhibiting virus replication ^40^. Functional studies initiated solely by this predicted PPI showed that human OASL binds to dsRNA to enhance RIG-I signaling, and that boosting OASL can help inhibit viral infection ^40^. Using novel PPIs predicted with HiPPIP, we could explain the apparent discordance between modern and historical genetic basis of schizophrenia ^41^, and the role of cilia in the pathogenesis of congenital heart disease ^42^. PPIs predicted by our method revealed a molecular basis for the negative association between schizophrenia and rheumatoid arthritis ^43^. These successes demonstrate that there is enormous potential for biomedical discovery buried in the largely-unexplored novel PPIs in the human interactome.

In this work, we applied the HiPPIP model to discover novel PPIs of IFITM1 and IFITM3, to potentially accelerate the discovery of the mechanism by which they inhibit ZIKV and other viral infections.

## Methods

We assembled the PPIs of IFITM1 and IFITM3 (‘IFITM interactome’) by predicting novel PPIs with HiPPIP ^39^ and collecting known PPIs from the Human Protein Reference Database ( HPRD) ^44^ and Biological General Repository for Interaction Datasets ( BioGRID) ^45^. HiPPIP uses a score cut-off of 0.5 to achieve a high precision of 97.5%, albeit successfully predicting only a few PPIs (recall of 5%), when evaluated on a held-out test data. Thus, the novel PPIs predicted by HiPPIP are highly dependable to lead to successful experiments. Furthermore, predicted PPIs with scores ranging from 0.41 to 0.65 were experimentally validated and found to be true interacting pairs ^39^. The HiPPIP model was also computationally evaluated on hub proteins and showed a better performance when compared to Qi *et al*.’s model ^39, 46^. HiPPIP is configured to present protein-pairs achieving a score of greater than 0.5 to be novel PPIs. Additional information regarding experimental validation of predicted interactions and model evaluation are available in prior work ^39^.

IFITM interactome figure was created using Cytoscape ^47^. Pathways associated with proteins in the interactome were collected using Ingenuity Pathway Analysis® suite ( www.ingenuity.com). Gene Ontology (GO) terms enriched in the interactome were computed using the BiNGO plugin of Cytoscape ^48^.

## Results

**Figure 1.**
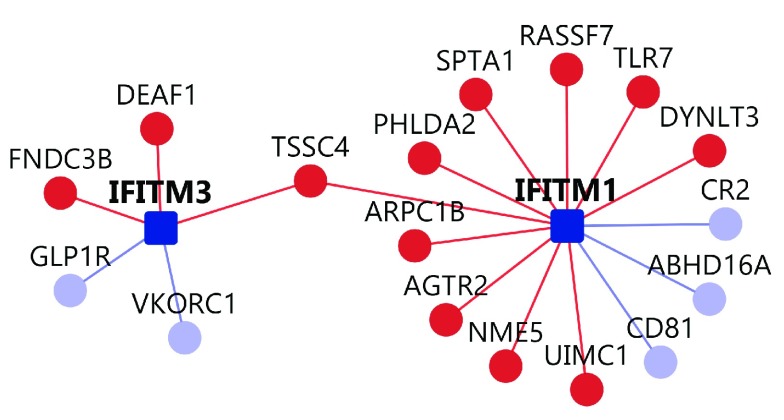
Protein-protein interactions (PPIs) of IFITM1 and IFITM3: Known PPIs were assembled from HPRD and BioGRID databases and novel PPIs were predicted using HiPPIP model. Novel interactors of IFITM1 and IFITM3 are shown as red colored nodes while previously known interactors are shown as light blue colored nodes. Novel interactors of IFITM1 and IFITM3 are shown as red colored nodes while previously known interactors are shown as light blue colored nodes. The known interactions are curated by ( HPRD) ^44^ and Biological General Repository for Interaction Datasets ( BioGRID) ^45^; any interactions that may be published in literature but not curated into these databases would not be seen here.

We assembled the PPIs of IFITM1 and IFITM3 (‘IFITM interactome’, Figure 1) by computing novel PPIs using the HiPPIP model and collecting known PPIs from publicly available databases. We found that the interactors of IFITMs are involved in relevant functions including immunity. Functional annotations of the novel interactors of IFITM1 and IFITM3, sourced from Wiki-Pi ^49^, revealed two main categories: development and innate immunity.

**Figure 2.**
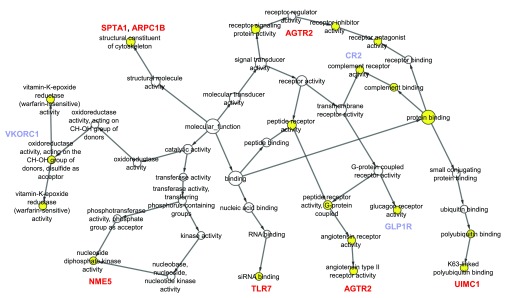
Gene Ontology terms enriched in the interactome of IFTIM1 and IFTIM3. Yellow color signifies statistically significant enrichments. Novel interactors that are associated with the GO terms are shown in red and known interactors in blue. See Table 2 for a list of terms associated with some selected genes.

Pathways associated with IFITM interactome computed with Ingenuity Pathway Analysis Suite® are given in Table 1. Gene Ontology biological process terms associated with the interactome, compiled with BiNGO ^48^ are shown in Figure 2 and Table 2.

**Table 1.** Pathways associated with IFITMs and their interactor. Pathway associations were computed with Ingenuity Pathway Analysis Suite®. Novel interactors are shown in bold.

Gene	Associated pathways
**AGTR2**	Gαi Signaling Renin-Angiotensin Signaling
**ARPC1B**	Axonal Guidance Signaling Signaling by Rho Family GTPases Actin Cytoskeleton Signaling Integrin Signaling Clathrin-mediated Endocytosis Signaling Ephrin Receptor Signaling RhoGDI Signaling Cdc42 Signaling Epithelial Adherens Junction Signaling RhoA Signaling CD28 Signaling in T Helper Cells fMLP Signaling in Neutrophils Rac Signaling Fcγ Receptor-mediated Phagocytosis in Macrophages and Monocytes Regulation of Actin-based Motility by Rho Remodeling of Epithelial Adherens Junctions Actin Nucleation by ARP-WASP Complex
CD81,CR2	PI3K Signaling in B Lymphocytes
CR2	IL-8 Signaling NF-κB Activation by Viruses Complement System
GLP1R	Gαs Signaling GPCR-Mediated Integration of Enteroendocrine Signaling Exemplified by an L Cell G-Protein Coupled Receptor Signaling
**AGTR2**	cAMP-mediated signaling
IFITM3, IFITM1	Interferon Signaling
**NME5**	Salvage Pathways of Pyrimidine Ribonucleotides Pyrimidine Ribonucleotides *De Novo* Biosynthesis Pyrimidine Ribonucleotides Interconversion Pyrimidine Deoxyribonucleotides *De Novo* Biosynthesis I
**SPTA1**	Sertoli Cell-Sertoli Cell Junction Signaling
**TLR7**	Role of Macrophages Fibroblasts and Endothelial Cells in Rheumatoid Arthritis Colorectal Cancer Metastasis Signaling Systemic Lupus Erythematosus Signaling NF-κB Signaling Role of Pattern Recognition Receptors in Recognition of Bacteria and Viruses phagosome formation Communication between Innate and Adaptive Immune Cells Crosstalk between Dendritic Cells and Natural Killer Cells Altered T Cell and B Cell Signaling in Rheumatoid Arthritis TREM1 Signaling Toll-like Receptor Signaling
**UIMC1**	Role of BRCA1 in DNA Damage Response

**Table 2.** Gene Ontology Biological Process terms associated with interactors. Novel interactors are shown in bold.

Interactor	Gene Ontology Terms
**AGTR2**	Angiotensin receptor activity Angiotensin type ii receptor activity Receptor antagonist activity Receptor inhibitor activity Glucagon receptor activity Peptide receptor activity, G-protein coupled Peptide receptor activity Receptor signaling protein activity
**ARPC1B**	Structural constituent of cytoskeleton
CR2	Complement receptor activity Complement binding
**DEAF1**	Neural Tube Closure Regulation Of Transcription From RNA Polymerase II Promoter Transcription From RNA Polymerase II Promoter Germ Cell Development Visual Learning Anatomical Structure Morphogenesis Regulation Of Mammary Gland Epithelial Cell Proliferation Negative Regulation Of Transcription, DNA-templated Positive Regulation Of Transcription, DNA-templated Embryonic Skeletal System Development
**NME5**	Nucleoside diphosphate kinase activity
**SPTA1**	Structural constituent of cytoskeleton
**TLR7**	siRNA binding
**UIMC1**	K63-linked polyubiquitin binding Polyubiquitin binding
VKORC1	Oxidoreductase activity, acting on the CH-OH group of donors, disulfide as acceptor Vitamin-K-epoxide reductase (warfarin-sensitive) activity Vitamin-K-epoxide reductase (warfarin-insensitive) activity

DEAF1, which encodes for deformed epidermal autoregulatory factor 1 homolog, is a transcription factor that binds to TTCG elements in DNA and is involved in neural tube closure, embryonic skeletal development and anatomic structure morphogenesis, and other functions ^50^. Additionally, DEAF1 is preferentially expressed in the CNS, particularly during early embryonic development. Mutations in DEAF1 have been associated withintellectual disability ^51^. A study involving Sendai-virus infected mouse embryonic fibroblasts (MEFs) found that DEAF1-/- deficient mice had lower levels of IFN-beta mRNA compared to the wild type controls, along with lower mRNA levels of other markers of viral infection ^52^.

FNDC3B, a membrane protein, was found to be associated with heart rate, height and corneal structure through genome-wide association studies. While its own functions are unknown, its known interactors are involved in regulation of glial cell apoptotic process, regulation of ion transport (sodium, potassium, calcium) and several cardiac processes.

SPTA1 which encodes for Spectrin alpha, erythrocytic 1 is among the predicted interactors with functional annotations related to innate immunity. It is a molecular scaffold protein that is involved in linking the plasma membrane to the actin cytoskeleton, thereby guiding cell shape and other processes pertaining to the cell’s structure ^53^. It is also involved in neural functions of actin filament organization, neurite outgrowth and axon guidance. Considering that IFITM1 and IFITM3 regulate permeability of the cell membrane, it is plausible that SPTA1 is also involved in aiding the host cell’s architecture to impede infection. Another predicted interactor that could be related to the host cell’s defense against infection is RASSF7, which is localized to the microtubule organizing center. While its function is unknown, it has known interactions with proteins that are involved in cell proliferation in the brain, regulation of neuroblast proliferation, nervous system development, synaptic vesicle fusion to presynaptic membrane, and viral budding and assembly.

Other predicted interactors include TSSC4, TLR7, and ARPC1B. TSSC4 is predicted to interact with both IFITM1 and IFITM3. TSSC4’s functions are unknown but its known interactions suggest that it may be involved in viral penetration into host nucleus, protein import into nucleus and immune response signaling, among other processes. TLR7 is involved in several functions and pathways related to innate immunity. It belongs to the Toll-like receptor (TLR) protein family and is involved in the recognition of pathogen-associated molecular patterns (PAMPs), along with stimulating the innate immune response by producing cytokines ^54^. Its known interactions have cellular components related to membrane components and protein binding. ARPC1B is part of actin related protein 2/3 complex; its known interactions suggest that it may be involved in processes of neuronal development such as axonogensis and development, neuron differentiation, nervous system development, and in immune related processessuch as the innate immune response, regulation of the immune response, etc. These functional annotations are sourced from Wiki-Pi ^49^; for example, see DEAF1 at http://severus.dbmi.pitt.edu/schizo-pi/index.php/gene/view/10522.

There is only one study that presents altered gene expression under ZIKV infection available in Gene Expression Omnibus ^16^. The study with eight samples (four infected and four control samples) showed that the infection of human neural progenitor cells (hNPCs) with the virus caused increased cell death and cell-cycle dysregulation ^16^. We examined whether any of the interacting genes were differentially expressed in that study and found five genes that were differentially expressed with a small fold-change but with significant p-value (< 0.005) ( Table 2): CD81, NME5, and RASSF7 were found to be under-expressed and FNDC3B and UIMC1 were found to be over-expressed ( Table 3).

**Table 3.** Interacting genes that are differentially-expressed under Zika virus infection, along with fold-change and significant p-values.

Interactor	Log-2 Fold Change	p-value
FNDC3B	0.92	0.00005
NME5	-1.55	0.00005
RASSF7	-0.46	0.00215
UIMC1	0.73	0.00005
CD81	-0.28	0.00325

## Discussion

Our goal here is to release the novel PPIs of IFITMs that we could predict computationally, recognizing their importance in mediating immune response in ZIKV infection. These predictions were obtained by using an experimentally and computationally validated algorithm HiPPIP, and are estimated to be highly accurate. Novel PPIs of IFITM1 and IFITM3 shed light on possible mechanisms of host invasion adopted by Zika virus including suppression of the immune system and cognitive and birth defects following infection. Based on the predicted protein-protein interactions, and the functions of the interacting proteins, we formulated three hypotheses about mechanisms of Zika virus infection.

### Cilia dysfunction in ependymal cells may be associated with microcephaly induced by Zika virus

IFITM1 and three of the novel interactors (NME5, UIMC1 and DYNLT3) were found in cilia interactome (i.e. interactome of proteins of the organelle cilia), and seemed to be associated with processes that led to hydrocephalus, a condition frequently associated with ciliopathies, in which cerebrospinal fluid accumulate in the brain ventricle giving rise to an enlarged head, viz. dysfunction of ciliated ependymal cells in the brain (unpublished results) ^55^. The fact that multiciliated ependyma is infected by a wide variety of viruses, possess motile cilia, micro villi and aherens junctions and also mediate cellular infiltration into the CNS indicate that it may be functioning as an immunological barrier ^56, 57^. This may point at the possibility that the multiciliated ependymal functions as a barrier which is also susceptible to the mechanisms of host invasion adopted by Zika virus and that cilia dysfunction may be a potential mechanism. In a recent study, delay in cilia disassembly was identified to be the cause of premature differentiation of neural progenitor cells (NPCs) in the ventricular zone which led to microcephaly ^58^. In an earlier study, the same group linked premature differentiation of NPCs to infection by Zika virus and impaired brain cellularity and structural organization of the cortical plate that follows ^59^.

While it has been established that defects in adhesion of ependymal cells and formation of cilia lead to hydrocephaly, its shared mechanisms with microcephaly, within the context of Zika infection has not been studied extensively. Interestingly, it has been reported that radial glial cells (RGCs), that give rise to ependymal cells, and astrocytes are more susceptible to infection by Zika virus than neurons ^60^. Moreover, RGCs constitute a barrier between brain parenchyma- a site of Zika infection- and ventricles from the embryonic stage ^61^. Fetal mice infected with Zika virus exhibit a reduction in the size of lateral ventricles ^62^. It has also been suggested that genes implicated in microcephaly may also influence the development of hydrocephalus. For example, ASPM which has undergone rapid evolution over the course of recent evolution of hominids is implicated in the size of cerebral cortex and neurogenesis and is also associated with primary microcephaly ^63^. The identification of ASH domains in ASPM has indicated that it may also be implicated in cilia dysfunction and hydrocephalus ^64^. ASH domains are normally found in proteins that localize to cilia, such as Hydin and OCRL which are associated with microcephaly and Lowe oculocerebrorenal syndrome respectively ^65, 66^. In this respect, it has also been observed that injection of pregnant mice with Zika virus, leads to infection of RGCs that are responsible for cortex development located in the dorsal ventricular zone of fetal mice ^67^.

The expression of IFITM1 in endothelial cells of various organs such as the bladder, brain and stomach is correlated with the maturation of blood vessels ^68^. It is induced during maturation stages of angiogenesis in vitro while in vivo it is stably expressed by microvascular endothelial cells which are quiescent ^68^. A role for IFITM1 in formation of stable contacts between cells during lumen formation in endothelial tissue was revealed by a study. On knockdown of IFITM1, intercellular vacuoles failed to fuse and form a multicellular lumen due to the mislocalization of OCLN (occludin), which normally interacts with IFITM1 and localizes to tight junctions between endothelial cells ^68^. It was speculated that the mechanism allowing IFITM1 to regulate assembly of tight junctions may be related to endosomal trafficking since internalized OCLN is returned to the plasma membrane from recycling endosomes during remodeling of tight junctions in endothelial cells. IFITM1 has also been shown to function in the endosomal pathway to inhibit viral infection ^69^. Microcephaly may arise due to dysfunctional DNA repair systems that lead to increased apoptosis of neural progenitor cells having a low endurance for damage and under normal conditions, migrate to the cortical plate to form various structures in the brain ^70^. Depletion of RAB80 or UIMC1- a novel interactor of IFITM1- which occurs in a complex with p73 when overexpressed, impairs translocation of BRCA1 to DNA damage sites resulting in defective control of cell cycle and repair of double strand breaks ^71, 72^. p73 has been implicated in the development and maintenance of ependymal cells and in animals deficient in p73, increased apoptosis and lack of differentiation of RGCs into ependymal cells accompanied by loss of motile cilia resulting in hydrocephalus and hippocampal dysgenesis were reported ^73, 74^. NME5 is highly expressed in ependymal cells and moderate to marked hydrocephalus along with ciliary dysfunction has been observed in mice homozygous for NME5 ^75^. It is known that patients with HSV-2 (Herpes simplex virus-2) infection are more susceptible to the teratogenic effects induced by Zika virus, since HSV-2 infection enhances the sensitivity of placental tissue which facilitates the entry of Zika virus into cells mediated by TAM receptors that recognize pathogen associated molecular patterns (PAMPs) ^76, 77^. It has been shown that VP26, the capsid surface protein of HSV, interacts with DYNLT3 which is a component of the motor protein, cytoplasmic dynein 1, mediating retrograde movement of vesicles and organelles along microtubules within cells ^78^.

RASSF7 (another novel interactor of IFITM1), has been shown to directly interact with DISC1 whose role in formation of astrocytes, which express primary cilia, in the embryonic brain via modulation of RAS/MEK/ERK signaling has been revealed in a study ^79, 80^. RASSF7 is a centrosomal protein that regulates microtubule dynamics, the knockdown of which in *Xenopus* has been reported to cause nuclear breakdown, apoptosis and loss of tissue architecture in the neural tube ^81^. One of the novel interactors predicted for IFITM3 is DEAF1, mutations in which has been linked to white matter disease, microcephaly and syndromic intellectual diability using whole exome sequencing ^82^. Exencephaly has been observed in mice homozygous for DEAF1 which is involved in the development of the neural tube ^83^. TSSC4 is associated with Beckwith Wiedemann syndrome which is incidentally also characterized by microcephaly in addition to other physical manifestations and interacts with CEP76, a candidate gene associated with autosomal recessive congenital microcephaly and found in ciliated cells ^84– 86^. The novel interactor PHLDA2 is also associated with Beckwith Wiedemann syndrome ^87^.

### Inhibitory action of IFITM1 may be mediated by effector functions of natural killer cells which may also be targeted for immunosuppression

The novel interactions of TLR7 and CD81 with IFITM1 opens up the possibility that the inhibitory action of IFITM1 on Zika virus may be mediated by effector functions of natural killer (NK) cells. At the same time, it has been shown that Zika viruses evade anti-viral responses by inhibiting interferon-mediated signaling ^88^. So, the novel interactions of TLR7 and CD81 with IFITM1 may also serve as potential targets of Zika virus by which it suppresses immune responses of the host.

NK cells when activated combat infection by Zika virus ^89^. TLR7 (a novel interactor predicted for IFITM1) recognizes viral RNA in endosomes and mounts defense mechanisms against flaviviruses including JEV (Japanese encephalitis virus) by activating NK cells to stimulate secretion of pro-inflammatory cytokines, increase microglial activation and infiltration of peripheral immune cells in the brain ^90, 91^. Mice with TLR7 knocked out have been shown to be more susceptible to infection by JEV ^92^. Another novel interactor of IFITM1- CD81 inhibits functions of NK cells when cross linked with HCV (Hepatitis C virus) and associates with GRP56 which negatively regulates effector responses of NK-92 cells dependent on IL-2 signaling including secretion of pro-inflammatory cytokines and cytolytic proteins ^93^. Also, malformations associated with microcephaly syndrome have been reported in patients with null GPR56 ^94^.

### Proteins that influence cytoskeletal events may be targeted by Zika virus to facilitate host invasion

Infection by Zika virus has been known to cause drastic re-organization of cytoskeletal structures surrounding replication factories in the cell, which are regions of the endoplasmic reticulum re-modeled following viral infection to allow viral replication ^95^. The novel interactions of FNDC3B with IFITM3 and SPTA1, ARPC1B and AGTR2 with IFITM1 may shed light on mechanisms of host invasion underlying cytoskeletal re-arrangements that follow Zika virus infection.

FNDC3B- a novel interactor of IFITM3 promotes cell migration by cooperating with ANXA2 ^96^. FNDC3B has been found to be upregulated in samples of hepatocellular carcinoma infected with HBV (Hepatitis B virus) or HCV and overexpressed in normal liver tissue infected with viral hepatitis ^97^. It has also been reported that the expression of FNDC3B increases on induction of GDF-15 which is known to regulate expression of genes that orchestrate changes in cytoskeleton and adhesion junctions ^97^. In this respect, it is interesting to note that IFITM3 has been suggested to inhibit early stages of Zika virus infection by directly altering the properties of cell and/or viral membranes and blocking formation of fusion pores ^98^. SPTA1 is associated with spinocerebellar ataxia which also has microcephaly as one of its physical manifestations ^99^. Spectrins form complexes with intracellular networks by interacting with actin, ankyrin and adducin ^99^. Deletion of adducin gives rise to lethal hydrocephalus in mice ^100^. Spectrins influence cell adhesion and spreading and are also found in the cortical cytoplasm of ependymal cells ^99^. It has been reported that OSGEP and TP53RK, both of which are implicated in nephrotic syndrome with primary microcephaly, interact with components of the ARP2/3 complex including ARPC1B involved in actin remodeling at lamellipodia ^101, 102^. Lesions in white matter found in elderly people have been associated with polymorphisms in AGTR2, a novel interactor of IFITM1and an antagonist of AGTR1, which influences angiogenesis induced by VEGF ^103^. Cytoskeletal events guided by VEGF signaling are known to orchestrate formation of vascular lumen, which might contribute to the development of microcephaly ^104^.

## Other resources

See http://severus.dbmi.pitt.edu/schizo-pi or http://severus.dbmi.pitt.edu/wiki-pi for annotations compiled from various databases for each of the individual proteins.

## Data availability

All pertaining data are provided in the manuscript.
