# Supplementary material for: Predicted protein interactions of IFITMs may shed light on mechanisms of Zika virus-induced microcephaly and host invasion
Source: F1000Res. 2017 Nov 21;5:1919. Originally published 2016 Aug 5. [Version 2] doi: 10.12688/f1000research.9364.2 (PMC5747333; doi:10.12688/f1000research.9364.2)
Supplement: Peer review history

# Peer review: Referee response for version 2

**DOI:** 10.5256/f1000research.13765.r28220

**Published:** 2018-01-02

**Authors:** Nicholas Eyre

**Affiliations:** 1 School of Biological Sciences, University of Adelaide, Adelaide, SA, Australia

The changes included in this revised manuscript somewhat improve the description of the HiPPiP model and add some useful (albeit highly speculative) discussion of the potential mechanisms of IFITM antiviral activities against ZIKV. However, I am still not entirely convinced of the validity of the HiPPiP model and some of the the statements supporting this model appear to be exaggerated (e.g. *"Thus, the novel PPIs predicted by HiPPIP are highly dependable to lead to successful experiments"* - **I am not sure what this means, and *"Furthermore, predicted PPIs with scores ranging from 0.41 to 0.65 were experimentally validated and found to be true interacting pairs"* - I am not convinced about the experimental validation detailed in this prior publication as the presented immunoprecipitation data does not include appropriate negative controls, molecular weight markers, details about antibody sources or specificity etc.). Future studies of this nature would be greatly strengthened by experimental validation of computationally predicted protein-protein interactions and exploration of the importance of the candidate host factors for the ZIKV replication cycle (e.g. via knockdown / knockout and/or pharmacological targeting of the candidate host factor and examination of effects on the ZIKV replication cycle).

I have read this submission. I believe that I have an appropriate level of expertise to confirm that it is of an acceptable scientific standard, however I have significant reservations, as outlined above.

# Peer review: Referee response for version 2

**DOI:** 10.5256/f1000research.13765.r28662

**Published:** 2017-12-20

**Authors:** Rama Rao Damerla

**Affiliations:** 1 Memorial Sloan Kettering Cancer Center, New York, NY, USA

The manuscript in review uses a previously tested model of protein-protein interaction (PPI) called High-confidence Protein-Protein Interaction Prediction (HiPPIP) to predict novel PPIs of two small membrane-associated interferon-inducible transmembrane proteins IFITM1 and IFITM3. The authors cite previously reported data on the role of IFITM1 and IFITM3 in inhibiting Zika virus (ZIKV) and make a valid argument that the novel PPIs of IFITM1 and IFITM3 from the HiPPIP model could possibly predict mechanisms of host invasion and immune suppression by Zika virus (ZIKV) and members of its family *Flaviviridae*. They also analyze functional and pathway associations of the interacting proteins and discuss possible roles of these proteins as targets for ZIKV infection. The title, abstract and article are clearly presented and make an engaging read. Although the authors present an extensive discussion on novel interactors, the article ends arbitrarily. A conclusion paragraph summarizing the results and presenting new ideas for future research would be apt.

I have read this submission. I believe that I have an appropriate level of expertise to confirm that it is of an acceptable scientific standard.

# Peer review: Referee response for version 2

**DOI:** 10.5256/f1000research.13765.r28221

**Published:** 2017-11-22

**Authors:** Judith Klein-Seetharaman

**Affiliations:** 1 Division of Metabolic and Vascular Health, University of Warwick, Warwick, UK

Looks good

I have read this submission. I believe that I have an appropriate level of expertise to confirm that it is of an acceptable scientific standard.

# Peer review: Referee response for version 1

**DOI:** 10.5256/f1000research.10083.r17282

**Published:** 2017-01-12

**Authors:** Sandeep Chakraborty

**Affiliations:** 1 Plant Sciences Department, University of California, Davis, CA, USA

This manuscript presents a use model of the a protein-protein interaction method developed by the author along with other collaborators, focused on a very important pathogen (Zika) in the present circumstances. It is lucidly written and well presented.

 The biggest shortcoming of this manuscript is the failure to cite several previous work related to the interferon-inducible transmembrane protein 3 ("The Antiviral Effector IFITM3 Disrupts Intracellular Cholesterol Homeostasis to Block Viral Entry" - Amini-Bavil-Olyaee, et. al, 2013, "The antiviral effector IFITM3 disrupts intracellular cholesterol homeostasis to block viral entry", Amini-Bavil-Olyaee, et. al, 2013 to name a couple).

 Furthermore, the current manuscript and its stated methodology does not find two proteins (VAPA and OSBP) that have been shown to have interactions with IFITM3. This would have been a clincher. It would also be proper to mention other work related to IFITM mechanism ("IFITM Proteins Restrict Viral Membrane Hemifusion": Li et. al, 2013) - and focus on the viral membrane hemifusion mentioned there.

 Minor comments:

1. "functional studies initiated solely" - capitalize functional.
2. Widespread use of "we" with only a single author.
3. "Computationally discovered PPIs have been shown to be highly accurate by computational evaluations and experimental validations of a few PPIs'." - overstated and speculative.
4. It is also speculative to correlate the expression changes of predicted interactors to these IFITMs, since there can be multiple other reasons for such expression changes ("whether any of the interacting genes were differentially expressed in that study and found five genes that were differentially expressed").

I have read this submission. I believe that I have an appropriate level of expertise to state that I do not consider it to be of an acceptable scientific standard, for reasons outlined above.

# Peer review: Referee response for version 1

**DOI:** 10.5256/f1000research.10083.r17909

**Published:** 2016-12-08

**Authors:** Nicholas Eyre

**Affiliations:** 1 School of Biological Sciences, University of Adelaide, Adelaide, SA, Australia

This manuscript reports a number of novel protein interactions of IFITM1 and IFITM3 proteins, as determined using a 'High-confidence Protein-Protein Interaction Prediction (HiPPIP)' computational prediction model of protein-protein interactions. Analysis of functional and pathway associations of the putative interacting proteins is also reported, as they may relate to IFITM-mediated restriction of Zika virus infection. The manuscript is generally well-written and the title is appropriate. However, there are several ways in which the study and manuscript could be improved:

1. The report is largely based upon the predictions generated by the HiPPIP model. As this model is not well-described here and has not been extensively validated experimentally in the literature, it would be helpful if the criteria/rules employed by the model were described (Are predictions based purely on structural features? Does the model take into account protein localization, regulation, tissue distribution, known interactions etc.?). In the Methods section it is simply stated that 'Computationally discovered PPIs have been shown to be highly accurate by computational evaluations and experimental validations of a few PPIs'. This statement is not well-justified and may be misleading. In support of the HiPPIP model, the author refers to the successful prediction of OASL interaction with RIG-I ('Functional studies initiated solely by this predicted PPI showed...'). However in the cited publication (on which Dr. Ganapathiraju is an author) ^1^ the HiPPIP model (and associated publications) was not referred to. The HiPPIP model should be more clearly described here, including some analysis/discussion of its success rate in predicting protein-protein interactions that have been experimentally validated.
2. As this report focuses on IFITM1 and IFITM3 antiviral functions and predicted interactions, it would be useful if the features and properties of these proteins were at least briefly described (domains, membrane topology, post-translational modifications, sub-cellular localization, tissue distribution and regulation). This would help to interpret the significance of the predicted interactions.
3. In the Results and Discussion some of the roles/properties of the predicted interacting partners are listed. It would be helpful if references were provided for these functions/properties (even if only to reviews). Furthermore, it would be helpful if these properties were discussed in the context of Zika virus and/or IFITM biology (e.g. commonalities in tissue/sub-cellular distribution and/or regulation and features of these proteins [e.g. domains] that may support their predicted interactions).
4. The manuscript should be carefully edited to clarify interactions that are purely predicted and not supported by experimental evidence (e.g. statements like 'TSSC4 interacts with IFITM1 and IFITM3' are misleading).
5. A conclusion/summary paragraph that briefly summarizes the major findings and their significance and potential future directions may be appropriate.   

I have read this submission. I believe that I have an appropriate level of expertise to confirm that it is of an acceptable scientific standard, however I have significant reservations, as outlined above.

## References

[1] ZhuJZhangYGhoshACuevasRAForeroADharJIbsenMSSchmid-BurgkJLSchmidtTGanapathirajuMKFujitaTHartmannRBarikSHornungVCoyneCBSarkarSN: Antiviral activity of human OASL protein is mediated by enhancing signaling of the RIG-I RNA sensor. *Immunity*.2014;40(6) : 10.1016/j.immuni.2014.05.007 936-48 10.1016/j.immuni.2014.05.007 24931123PMC4101812

# Peer review: Referee response for version 1

**DOI:** 10.5256/f1000research.10083.r15557

**Published:** 2016-08-08

**Authors:** Judith Klein-Seetharaman

**Affiliations:** 1 Division of Metabolic and Vascular Health, University of Warwick, Warwick, UK

The title, abstract and article overall are well written and clear. The design, methods and analysis are mostly well described, although some detail could be added. In particular, given that the IFITM interactome contains a large number of previously unknown PPI’s, it would be useful to give a little more detail on the methodology on how these predictions were obtained, rather than just stating that HiPPIP was used. In particular, it would be useful to understand what cut-off was used and a brief mentioning of the prediction methodology in general. An estimate of false positive and false negative errors for the prediction in Figure 1 would be particularly helpful.

 In the analysis, the legend for Figure 2 needs expansion. It is not clear what the edges signify and in particular what is the meaning of directionality in the arrows.

 I object to the wording used on page 2 “which was validated to be a true PPI”, as any PPI evidence is debatable. I would reword to “which was experimentally validated”.

 I also object to the wording used on page 2 “Computationally discovered PPIs have been shown to be highly accurate”. Such a blanket statement is clearly not true, as there are many predictions out there that are highly inaccurate. A more specific example needs to be provided here.

 A minor suggestion is to replace “HiPPIP model” with “the HiPPIP model”.

I have read this submission. I believe that I have an appropriate level of expertise to confirm that it is of an acceptable scientific standard.
